# Analysis of regional agricultural carbon emission efficiency and influencing factors: Case study of Hubei Province in China

**DOI:** 10.1371/journal.pone.0266172

**Published:** 2022-04-28

**Authors:** Tengyu Shan, Yuxiang Xia, Chun Hu, Shunxi Zhang, Jinghan Zhang, Yaodong Xiao, Fangfang Dan

**Affiliations:** School of Chemistry and Environmental Engineering, Wuhan Polytechnic University, Wuhan, China; Szechenyi Istvan University: Szechenyi Istvan Egyetem, HUNGARY

## Abstract

In recent years, China’s industrial economy has grown rapidly and steadily. Concurrently, carbon emissions have gradually increased, among which agricultural production is an important source of greenhouse gas emissions. It is necessary to reduce agricultural carbon emissions by improving their efficiency to achieve the global goal of peak carbon dioxide emissions in 2030. From a dynamic and static point of view, this study puts agricultural carbon emissions into the evaluation index system of agricultural carbon emission efficiency and analyzes the agricultural carbon emission efficiency and its influencing factors in Hubei Province. First, the unexpected output Slacks-based measure (SBM) model in data envelopment analysis was used to evaluate the agricultural carbon emission efficiency of Hubei Province in 2018 and compared it with other provinces horizontally. Second, the Malmquist–Luenberger index was used to analyze the comprehensive efficiency of agricultural carbon emissions in Hubei Province from 2004 to 2018. The role of technological progress and technical efficiency change in the development of low-carbon agriculture in Hubei Province was analyzed. The results showed that agricultural production efficiency in Hubei Province improved from 2004 to 2018, and the overall level was slightly higher than the average level in China. However, agriculture has not eliminated the extensive development modes of high input, low efficiency, high emission, and high pollution. The efficiency of technological progress in agricultural resource utilization in Hubei Province was close to the optimal level. The improvement space was small. Hence, the low efficiency of agricultural technology is a key factor restricting the improvement of agricultural production efficiency. The results provide a reference for low-carbon agricultural policy formulation and expand the policy choice path. This has practical significance.

## Introduction

In recent years, anthropogenic activities have led to many problems caused by climate change. These problems have gradually become a focus of the international community. In December 2015, 196 parties adopted the Paris Agreement at the Paris Climate Change Conference. Its goal is to limit global warming to well below 2, preferably to 1.5 degrees Celsius, compared to pre-industrial levels. China adheres to the green development path of ecological priority and low-carbon emissions, and actively responds to climate change. The Party Central Committee proposed bringing down peak carbon dioxide emissions and strive toward carbon neutrality as the overall layout of an ecological civilization. China is trying to peak carbon emissions by 2030, and achieving carbon neutrality before 2060 [1]. Agriculture is not only an important global greenhouse gas emission source but also a huge carbon sink system [2]. Low-carbon agriculture emphasizes the reduction of the overall agricultural energy consumption and carbon emissions. It should advocate for minimal use of chemical fertilizers and pesticides, and more use of organic fertilizer and renewable energy as ecological agriculture does. It should also pay more attention to the reduction of overall agricultural energy consumption and carbon emissions in the case of increasing agricultural energy consumption [3]. An agroforestry system that deliberately integrates trees and crops with livestock in agricultural production could potentially increase carbon sequestration and decrease greenhouse gas emissions from terrestrial ecosystems, thus mitigating global climate change [4]. Under the background of increasingly severe global warming, China––as a traditional agricultural country––advocates for low-carbon agriculture as the inevitable choice to realize the harmonious development of economic growth and the ecological environment and promote sustainable development of agriculture. Agricultural emission reduction and carbon sinks are important means to achieve the peak potential of carbon emissions. Therefore, maximizing the efficiency of agricultural carbon emissions and minimizing the generation of agricultural greenhouse gases under the premise of limited resources are the most important problems. Related research on agricultural carbon emissions is particularly necessary.

At present, research on agricultural carbon emissions has gradually evolved, mainly focusing on the analysis of influencing factors of carbon emissions [5, 6], the temporal and spatial distribution of agricultural carbon emission intensity, and regional differences [7, 8], research on the influencing factors and spillover effects of agricultural carbon emissions efficiency [9], and research on the relationship between agricultural economic development and agricultural carbon emissions using the TAPIO decoupling model [10, 11]. Katarzyna et.al [12] described renewable energy sources were ones that replenish (or renew) themselves naturally and researched the development of the wind energy sector, witch was one of the basic renewable energy sources, in 28 European Union countries. They believed that the use of renewable energy such as wind energy has effectively promoted the reduction of carbon emissions in Europe. Zhongying Hu [13] combine with other factors, such as agricultural development level, agricultural industrial structure and environmental regulation, builds a dynamic panel model and applies the GMM to make an empiri-cal study of their effect on agricultural carbon emissions. Szilard CZOBEL et.al [14] researched the seasonal and inter-annual dynamics of the stand level CO_2_-flux and production of sandy grassland that has been extensively grazed for decades by performing field investigation and using portable, non-destructive own developed chambers and infrared gas analyses. By constructing the calculation system of carbon emissions of planting industry, Xiaowen Dai et.al [15] analyzed the changes in driving factors of carbon emissions of planting industry in different regions of China by using the logarithmic mean divisor index (LMDI) on the basis of scientific calculation. Bioethanol production by-product processing technology can play a role in exchanging imported feed and can ensure that the local population of ruminants is immune to bovine spongiform encephalopathy (BSE) [16].Under unfavorable field conditions, the development, and trends of growth and nutrient content parameters of three different plant species as green manure secondary crops were studied as functions of two different fertilizer doses [17]. Jianxiang Tian estimated the carbon emissions from agricultural production in Hunan Province from 1998 to 2012 and analyzed the trend of agricultural carbon emissions in Hunan Province and the decoupling relationship between carbon emissions and agricultural output [18]. Studies that analyzed the factors influencing agricultural carbon emissions include: Brett A. Bryan quantified the emissions from agriculture, based on the typical efficiency assessment when only two objectives are considered, efficiency improvements involve significant unexpected trade-offs against other objectives and incur significant opportunity costs [19]. Appiah Kingsley studied the decomposition of agricultural production into crop production and livestock production to elucidate the contribution of each variable to CO_2_ emissions. The empirical results show that a 1% increase in economic growth rate, crop production index and livestock production index will lead to a corresponding increase in CO_2_ emissions by 17%, 28% and 28%. Accordingly, the direction of causality between variables was examined using PMG estimator [20]. Berna Aydoğan study examined the dynamic relationship between carbon dioxide emissions per capita, economic growth, and agricultural value added, where non-renewable energy consumption was negatively correlated with agricultural value added, while carbon dioxide emissions were negatively correlated with the square of real GDP and renewable energy consumption [21]. Zaim et al [22], Zofio et al [23], and Zhou et al [24] evaluated the efficiency of CO_2_ emissions in various countries and regions using different methods from the DEA model, respectively.

Currently, the related research on agricultural carbon emissions in Hubei Province is relatively scattered. Therefore, considering that Hubei Province is leading the construction, operation, and maintenance of the carbon emission registration system in the national carbon market, this study aims to establish a systematic and scientific agricultural carbon emission measurement system according to the overall agricultural development and conduct in-depth research on the efficiency of agricultural carbon emissions in the province. This study used the SBM model and Malmquist–Luenberger index in data envelopment analysis (DEA) to evaluate the agricultural carbon emission efficiency of Hubei Province and 31 other provinces in China from 2004 to 2018 from static and dynamic perspectives. It analyzed the role of technological progress and efficiency changes in the development of low-carbon agriculture in Hubei Province. On this basis, corresponding countermeasures and suggestions were put forward for the problems existing in the development of low-carbon agriculture in Hubei Province. The carbon emissions mentioned in this paper were converted to carbon dioxide equivalents to facilitate analysis and processing. Carbon dioxide equivalent is a measurement unit used to compare the emissions of different greenhouse gases, obtained by multiplying tons of the gas by its greenhouse effect value. This method can standardize the greenhouse effects of different greenhouse gases (GHGs).

## 1 Overview of research area

Hubei Province lies between 29 01’ 53 "-33 6’ 47" north latitude and 108 21’ 42 "-116 07’ 50" E longitude, in the middle of China, and the terrain is in the transition zone from the second step to the third step [25]. The topography of Hubei Province is high on three sides and low in the middle; it is an incomplete basin with a gap in the north and an opening in the south. There are various landforms including mountains, hills, and plains. Hubei Province located in the Hanjiang River and Yangtze River, is rich in water resources. It is also located in the subtropical monsoon region, with abundant sunshine and rain, and a pleasant climate. It is known as the province of thousands of lakes and the land of fish and rice, and is suitable for farming [26].

Hubei Province is a major agricultural province in China and is an important grain-producing area and agricultural production base. Agricultural modernization in this province is developing rapidly. The farming and fishing industries at Hubei have certain advantages compared to other provinces. According to the Statistical Bulletin of the National Economic and Social Development of Hubei Province in 2021, the province has a population of 5.775 million and a total land area of 18.559 million km2 by 2020. Agriculture, forestry, animal husbandry, and fishery earned 435.869 billion yuan, an increase of 0.3% over the previous year at comparable prices. Concurrently, the grain production capacity remained stable. The total grain output of the province was 2.72743 million tons, an increase of 0.1%, and it was stable at more than 50 billion kg for eight consecutive years. The planting area was 4645.27 thousand hectares, an increase of 0.8%.

## 2 Methodology and data sources

### 2.1 Calculation of agricultural carbon emissions

First, the carbon emission sources of the agricultural system were determined, and then the total carbon emissions of the agricultural system were calculated by multiplying the amount of each carbon emission source with the corresponding emission coefficient of each carbon emission source. The specific calculation formula is as follows.


$$E=\sum E_{i}=T_{i}\times \delta_{i}$$ (1)

Where *E* is the total carbon emissions of agriculture, *E*_*i*_ is the carbon emission of the carbon source, *T*_*i*_ is the consumption of each carbon source, and *δ*_*i*_ is the carbon emission coefficient of each carbon source.

### 2.2 Undesirable output SBM model

DEA has proven to be an effective tool for measuring the efficiency and productivity of similar decision-making units and has been widely used in the evaluation of productivity and efficiency of industries, cities, and regions [27]. The traditional DEA model includes radial and angular measurements. Radial refers to the reduction or amplification of the input or output in equal proportion to achieve effectiveness, and angle refers to the angle of the input or output. Therefore, the traditional DEA model cannot fully consider the slack problem of input and output, and the measured efficiency value is inaccurate or biased [28]. Tone proposed a non-radial and non-angle slacks-based measure model to solve this problem [29]. This model compensates for the shortcomings of traditional models and puts relaxation variables directly into the objective function. Conversely, it eliminates the influence of the unrelenting change of slack variables on efficiency evaluation and, simultaneously, provides a new idea for system evaluation with unexpected output indicators. The basic form of the SBM is as follows.


$$min\rho =\frac{1-\frac{1}{M}\sum_{m=1}^{M}\frac{S_{m}^{-}}{X_{m0}}}{1+\frac{1}{G+B}(\sum_{g=1}^{G}\frac{S_{g}^{+}}{y_{0}^{g}}+\sum_{b=1}^{B}\frac{S_{b}^{+}}{y_{0}^{b}})}$$ (2)

$$s.t.\left\{ \begin{array}{c} X_{0}=X\lambda +s^{-}\\ y_{0}^{g}=Y^{g}\lambda -s^{g}\\ y_{0}^{b}=Y^{b}\lambda +s^{b}\\ \lambda ,s^{-},s^{b},s^{g}\geq 0 \end{array}\right.$$ (3)

Where *s* represents the slack of the input and output and *λ* is the weight vector. The objective function strictly decreases *s*^−^, *s*^*b*^, and *s*^*g*^. The decision-making unit has M inputs (*X*_1_, *X*_2_,⋯,*X*_*M*_), G expected outputs ($y_{0}^{1},y_{0}^{2}{,\cdots ,y}_{0}^{g}$) and B unexpected outputs ($y_{0}^{1},y_{0}^{2}{,\cdots ,y}_{0}^{b}$), representing the slack variables of inputs, expected outputs, and unexpected outputs, respectively. For the decision-making unit to be evaluated, the decision-making unit is DEA-effective when *ρ* = 1. At this time, the input-output ratio of the evaluated unit is the best among all the evaluation units, and there is no input redundancy or output shortage. When the input factors remain unchanged, the output will no longer increase, which is called Pareto efficient. In contrast, the decision-making unit is non-DEA efficient during *ρ*<1, and it can be optimized by adjusting the input factors and undesirable outputs [30].

### 2.3 Malmquist–Luenberger index

The Malmquist–Luenberger (ML) index was developed based on the Malmquist index. The Malmquist index was first proposed by a Swedish economist, Sten Malmquist [31]. Färe et al. combined the index with DEA theory, making the Malmquist index widely used to measure the productivity of financial, industrial, medical, and other departments [32]. However, the impact of unexpected outputs on production efficiency was not considered. Chung et al. improved the traditional DEA model, applied the direction distance function with bad output to the Malmquist model, and proposed the Malmquist–Luenberger productivity index based on the direction distance function [29]. The ML index from time t to time t+1 is expressed as follows.


MLtt+1=[1+D→0t(Xt,ygt,ybt;gt)1+D→0t(Xt+1,ygt+1,ybt+1;gt+1)×1+D→0t+1(Xt,ygt,ybt;gt)1+D→0t(Xt+1,ygt+1,ybt+1;gt+1)]12 (4)

It can be seen that, the ML index from *t* to *t*+1 is the geometric average of the ML index at two times. The ML index can not only solve the problems of unexpected output but also dynamically analyze the efficiency changes and influencing factors of the evaluated unit. The measurement of this index is based on the frontier function of environmental production constructed by resource input, expected output, and unexpected output, which can comprehensively reflect the changes in technological progress and technical efficiency of decision-making units [30]. The ML index can be decomposed into the technological change index (Tech) and the efficiency change index (Effch). The relationship is as follows.


$$ML_{t}^{t+1}={Tech}_{t}^{t+1}\times {Effch}_{t}^{t+1}$$ (5)

Tech expresses the movement of decision-making units on the production front caused by technological progress in the two stages, reflecting the degree of change in production technology between the two periods. When *Tech* >1, it means that with time, the production technology is constantly improving, and there is innovation and technological progress. Effch reflects the degree to which the decision-making unit improves or reduces the production efficiency, which is represented by the catch-up of the decision-making unit on the production front. When *Effch*>1, the efficiency improved between the two periods, and technical management was effective [30].

### 2.4 Data sources

According to this investigation, the main sources of agricultural carbon emissions in Hubei are carbon emissions from rural living energy consumption, such as coal, refined oil, electricity (farming power), straw, firewood, and biogas. It also includes carbon emissions generated during production operations, such as irrigation, fertilization, weeding, insecticide application, plowing, using of plastic films, domestic garbage disposal, and metabolic activities of livestock [33]. Therefore, the data used in this study included the amount of chemical fertilizer, agricultural film, pesticide, agricultural diesel, and effective irrigation area. The amount of chemical fertilizer, agricultural film, pesticide, and diesel oil used shall be subject to the actual amount used in that year, the irrigated area of agricultural water shall be subject to the irrigated area of cultivated land in that year, and the middle plowing area shall be subject to the actual sown area in that year. Relevant information on agricultural carbon emission sources are presented in Table 1.

**Table 1:** Agricultural carbon emission source coefficient and corresponding index table.

Source of carbon emissions	Corresponding indicators	Carbon emission factor	Source of coefficient
Fertilizer	Fertilizer application rate	0.8956kg CE/kg	Oak Ridge National Laboratory, USA
Pesticide	Pesticide application rate	4.9341kg CE/kg	Oak Ridge National Laboratory, USA
Agricultural film	Agricultural film application rate	5.18kg CE/kg	Institute of Agricultural Resources and Ecological Environment, Nanjing Agricultural University
Agricultural diesel	Agricultural diesel application rate	0.5927kg CE/kg	Intergovernmental Panel on Climate Change, IPCC
Rice cultivation	Effective irrigated area	4225kg CE/ha	Intergovernmental Panel on Climate Change, IPCC
Land ploughing	Effective arable land area	312.6kg CE/km^2^	Intergovernmental Panel on Climate Change, IPCC
Intestinal fermentation of livestock	Annual pig feeding	25kg CE/year	Intergovernmental Panel on Climate Change, IPCC
	Annual cattle feeding	1358kg CE/year	Intergovernmental Panel on Climate Change, IPCC
	Annual sheep feeding	125kg CE /year	Intergovernmental Panel on Climate Change, IPCC
Livestock manure treatment	Annual pig feeding	233kg CE/year	Intergovernmental Panel on Climate Change, IPCC
	Annual cattle feeding	421kg CE/year	Intergovernmental Panel on Climate Change, IPCC
	Annual sheep feeding	102kg CE/year	Intergovernmental Panel on Climate Change, IPCC

## 3 Results and analysis

### 3.1 Agricultural carbon emissions in hubei province

**Fig 1:**
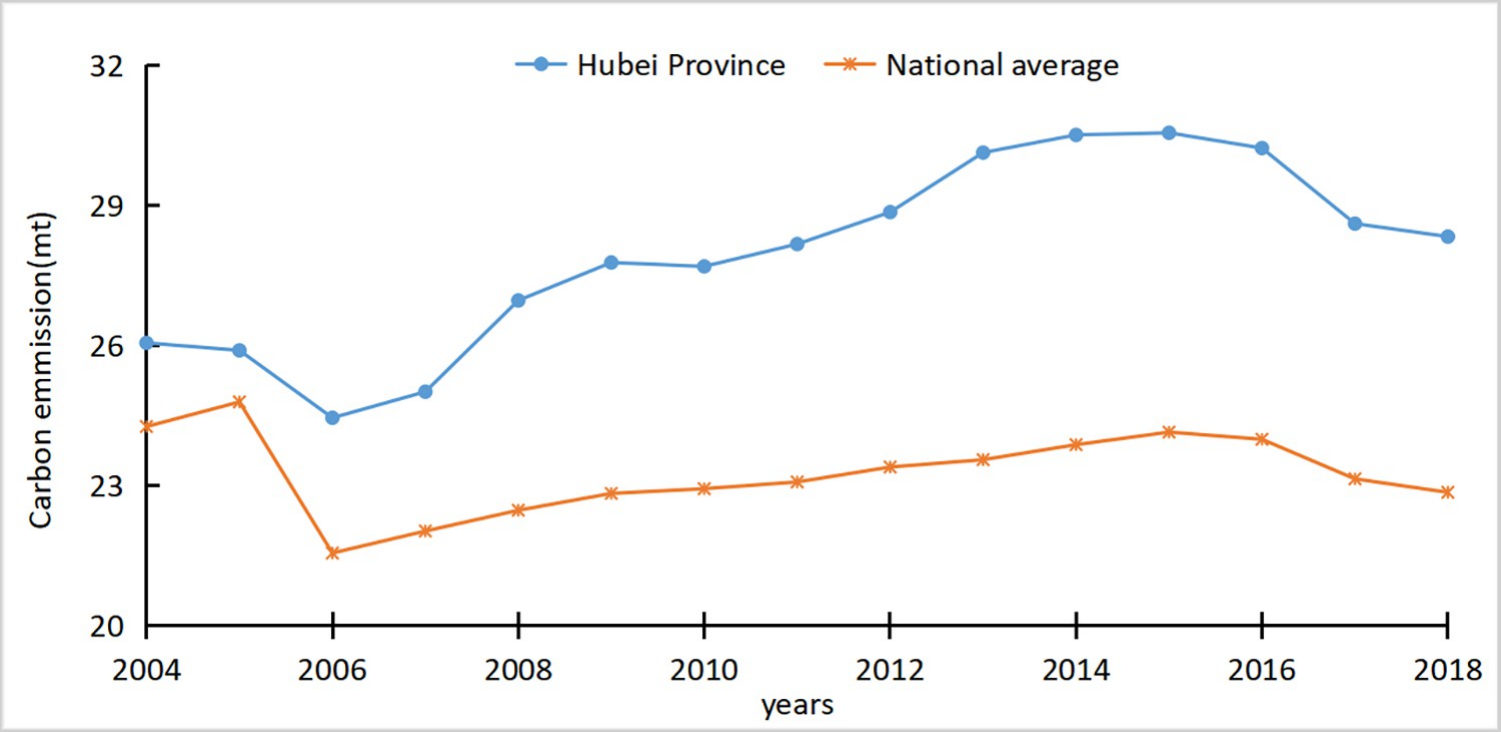
Hubei Province and national average agricultural carbon emission measurement trends.

The following were selected as agricultural carbon emission sources to calculate agricultural carbon emissions in Hubei Province from 2004 to 2018: amounts of chemical fertilizer, agricultural film, pesticide, agricultural diesel, effective irrigation area, effective arable land area, animal intestinal fermentation, and animal manure treatment. Simultaneously, to analyze the average agricultural carbon emissions of Hubei Province compared with other provinces, this study calculated the agricultural carbon emissions of all provinces and the average national agricultural carbon emissions, as shown in Fig 1.

The above figure reflects the changes in the average carbon emissions in Hubei Province and the national average from 2004 to 2018. The values of Hubei Province was generally lower than the national level, reaching 6.4 million tons in 2015 in terms of carbon emissions. From the perspective of development trends, both were relatively consistent and reached the lowest value in 2006, then slowly increased and reached the peak value in 2015, and then showed a downward trend. The changes in the past 15 years could be divided into two stages, with 2015 as the cut-off point. Before 2015, the average carbon emissions of Hubei Province and the whole country were on a rising state. During this period, extensive agricultural production was implemented throughout the country. To rapidly increase crop yield, problems such as improper land use, excessive application of pesticides and fertilizers, and environmental problems was caused by the neglect of resource utilization efficiency and excess energy. After 2015, the average agricultural carbon emissions in Hubei Province and the entire country dropped considerably. This was because after many countries adopted the Paris Agreement at the Paris Climate Change Conference in December 2015, China actively adopted several policies related to low-carbon agriculture and achieved remarkable emission reduction effects.

### 3.2 Efficiency evaluation of agricultural carbon emissions in Hubei province

Since the DEA method required the evaluated units to have high homogeneity and the agricultural production environment in Hubei Province changed greatly from to 2004–2018, it was impossible to directly analyze the agricultural carbon emission efficiency in Hubei Province from 2004 to 2018. Therefore, it was necessary to use the SBM model under the condition of variable returns to statically analyze the efficiency of agricultural carbon emissions in each province in 2018 to understand the current situation of carbon emission efficiency in Hubei Province. Using panel data from 2004 to 2018, the Malmquist–Luenberger index of each province was calculated to dynamically analyze the efficiency change of agricultural carbon emissions in Hubei Province.

#### 3.2.1 Static evaluation based on the unexpected output SBM model

**Fig 2:**
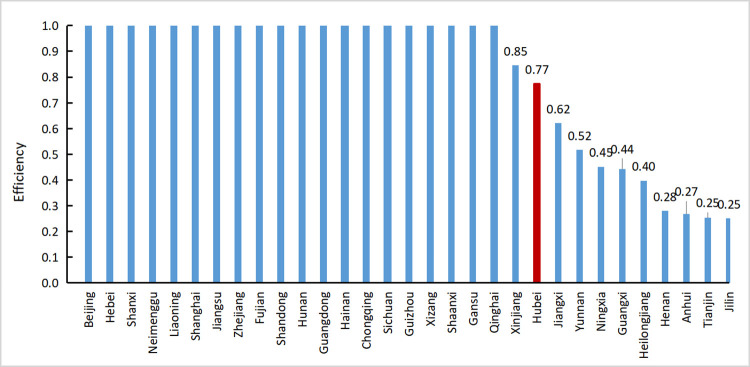
Ranking map of agricultural carbon emission efficiency of 31 provinces in 2018.

Based on a careful analysis of input and output in agricultural production activities, combined with three productivity factors, this study selected the number of employees in the primary industry, total sown area, total power of agricultural diesel, application amount of chemical fertilizers, and investment in fixed assets of agriculture, forestry, animal husbandry, and fishery as input indicators, and selected the total output value of agriculture, forestry, animal husbandry and fishery, afforestation area, and agricultural carbon emissions as output indicators to build an index system of agricultural carbon emission efficiency. The total output value and afforestation area were expected outputs, and agricultural carbon emissions were unexpected outputs. The efficiency of agricultural carbon emissions in 2018 was evaluated using the SBM model with unexpected output under variable returns to scale using 31 provinces as decision-making units. The calculation results are listed in Table 2. This study draws a bar chart according to the order of agricultural carbon emission efficiency from large to small, and the results are shown in Fig 2 to conveniently observe the agricultural carbon emission efficiency values of various provinces and cities and reflect the position of agricultural carbon emission efficiency in Hubei Province more intuitively.

**Table 2:** Agricultural carbon emission efficiency evaluation of 31 provinces in 2018.

DMU	Score	S-(1)	S-(2)	S-(3)	S-(4)	S-(5)	S+(1)	S+(2)	S+(3)
Beijing	1	0	0	0	0	0	0	0	0
Tianjin	0.27	0	275.35	184.56	7.26	110.40	104.46	0	31.46
Hebei	1	0	0	0	0	0	0	0	0
Shanxi	1	0	0	0	0	0	0	0	0
Neimenggu	1	0	0	0	0	0	0	0	0
Liaoning	1	0	0	0	0	0	0	0	0
Jilin	0.25	170.18	5235.56	2784.88	171.51	274.71	1388.29	0	0
Heilongjiang	0.44	0	10465.21	3237.32	50.20	301.60	2566.16	0	46.62
Shanghai	1	0	0	0	0	0	0	0	0
Jiangsu	1	0	0	0	0	0	0	0	0
Zhejiang	1	0	0	0	0	0	0	0	0
Anhui	0.28	615.24	6620.44	5060.55	175.47	318.29	1811.72	0	71.25
Fujian	1	0	0	0	0	0	0	0	0
Jiangxi	0.77	0	2291.62	393.73	0	47.59	452.72	0	0
Shandong	1	0	0	0	0	0	0	0	0
Henan	0.40	866.29	7325.61	4040.72	373.08	1500.65	2036.62	0	39.36
Hubei	0.85	0	2090.50	63.56	34.69	105.55	317.96	0	0
Hunan	1	0	0	0	0	0	0	0	0
Guangdong	1	0	0	0	0	0	0	0	0
Guangxi	0.45	464.24	2860.65	1918.71	86.37	657.86	850.46	0	0
Hainan	1	0	0	0	0	0	0	0	0
Chongqing	1	0	0	0	0	0	0	0	0
Sichuan	1	0	0	0	0	0	0	0	0
Guizhou	1	0	0	0	0	0	0	0	0
Yunnan	0.62	292.48	1911.76	137.66	37.67	713.07	1226.78	0	0
Xizang	1	0	0	0	0	0	0	0	0
Shaanxi	1	0	0	0	0	0	0	0	0
Gansu	1	0	0	0	0	0	0	0	0
Qinghai	1	0	0	0	0	0	0	0	0
Ningxia	0.52	49.44	815.45	310.22	24.37	14.56	65.41	0	0
Xinjiang	1	0	0	0	0	0	0	0	0

Note: DMUs decision-making units. S-(1), S-(2), S-(3), S-(4), and S-(5) are the number of employees in the primary industry, total sown area, total power of agricultural diesel, application amount of chemical fertilizers, and investment in fixed assets of agriculture, forestry, animal husbandry, and fishery, respectively. S+(1), S+(2), and S+(3) are the output slack of the gross output value of agriculture, forestry, animal husbandry, fishery, and forestation area agricultural carbon emissions, respectively.

Table 2 and Fig 2 show 10 provinces with ineffective agricultural carbon emissions DEA in 2018, including Hubei Province. The agricultural carbon emission efficiency of Hubei Province was 0.85, ranking 21st among 31 provinces. Therefore, improvement of agricultural carbon emission efficiency could be achieved. Specifically, there were investment redundancies in the planting area of crops, total power of agricultural diesel, application amount of chemical fertilizers, and investment amount of fixed assets in agriculture, forestry, animal husbandry, and fishery. The excessive input of crop-sown area reflected the low efficiency of agricultural land use in Hubei Province. The excessive amount of total power of agricultural diesel oil indicated that agricultural workers were excessively dependent on agricultural machinery or improper operations when engaging in agricultural production activities. Excessive application of chemical fertilizers indicated that agricultural workers were not in good command of crop planting technology and were overly dependent on chemical substances. The value of the fixed–asset investment in the agriculture, forestry, and fishery industries was too large, indicating that agricultural investment failed to achieve the expected benefits. Therefore, provinces with low utilization efficiency could be effectively planned by the DEA method to improve the agricultural carbon emission efficiency of the corresponding provinces.

**Fig 3:**
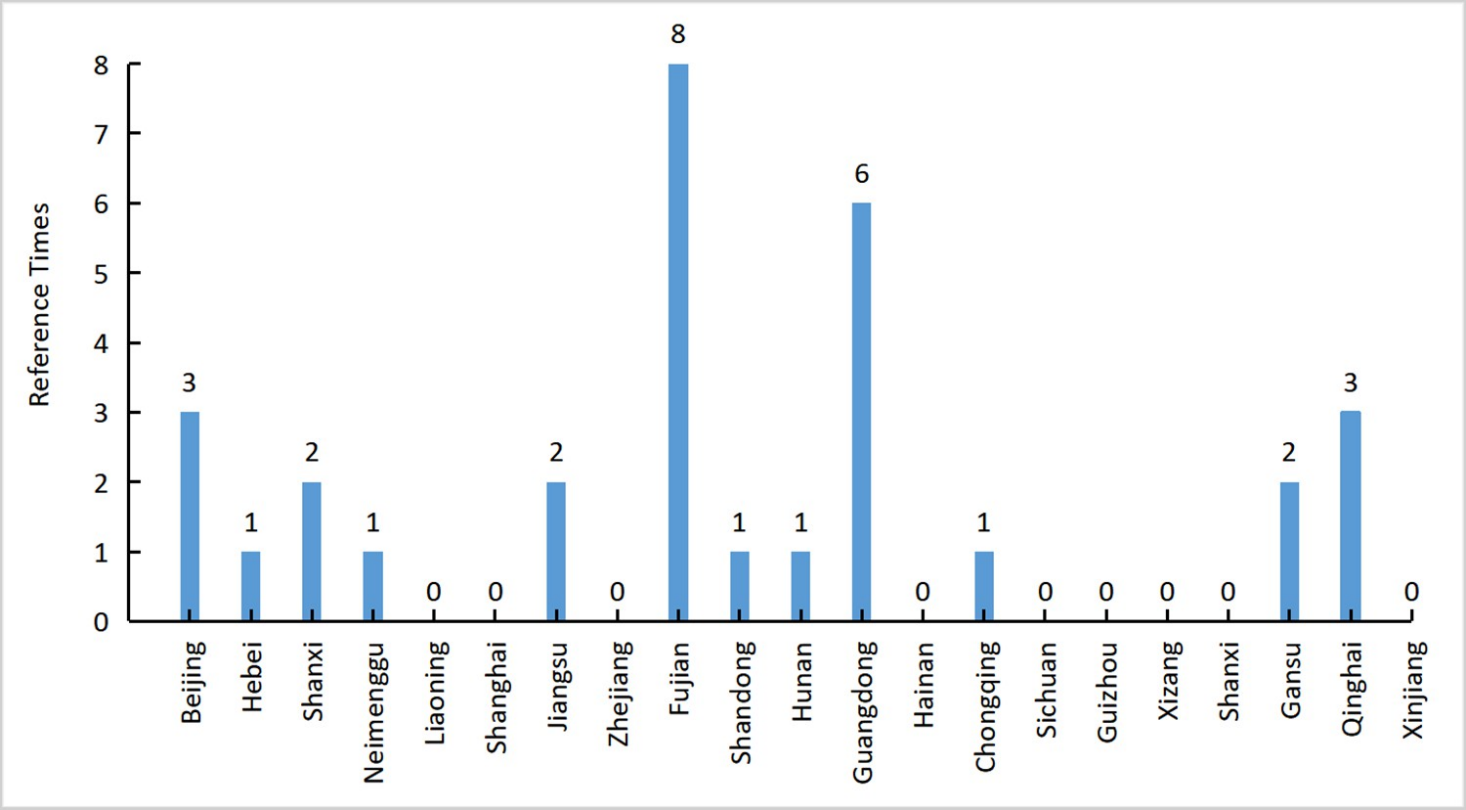
Reference times of high efficience DMU.

To further analyze the reference value of each decision-making unit (DMU), according to Fig 3, this study counted the reference times of high-efficiency DMUs and developed a histogram of the reference times of the decision-making unit with an efficiency value of 1 and other decision-making units. By counting this index, benchmark provinces with a relatively high agricultural production efficiency and a relatively low-carbon emission index can be found. The more times a DEA-effective province was referenced by other provinces, the greater the significance of low-carbon agricultural development in this province as a benchmark.

Fujian Province had been referred for eight times, followed by Guangdong Province which was referred for six times. This shows that the two provinces could be used as benchmarks for low-carbon agricultural production in China, and the development of low-carbon agriculture was relatively good. This could be used as a reference by other provinces and cities. The benchmark provinces and cities in Hubei Province included Hebei, Jiangsu, Fujian, and Guangdong provinces, to provide experience and reference for improving its carbon emission efficiency. In particular, in Jiangsu Province, which is also in the Yangtze River Economic Belt, the production environment and economic conditions of the two provinces were quite similar, with very high learning and reference values.

#### 3.2.2 Dynamic evaluation based on the Malmquist–Luenberger index

**Fig 4:**
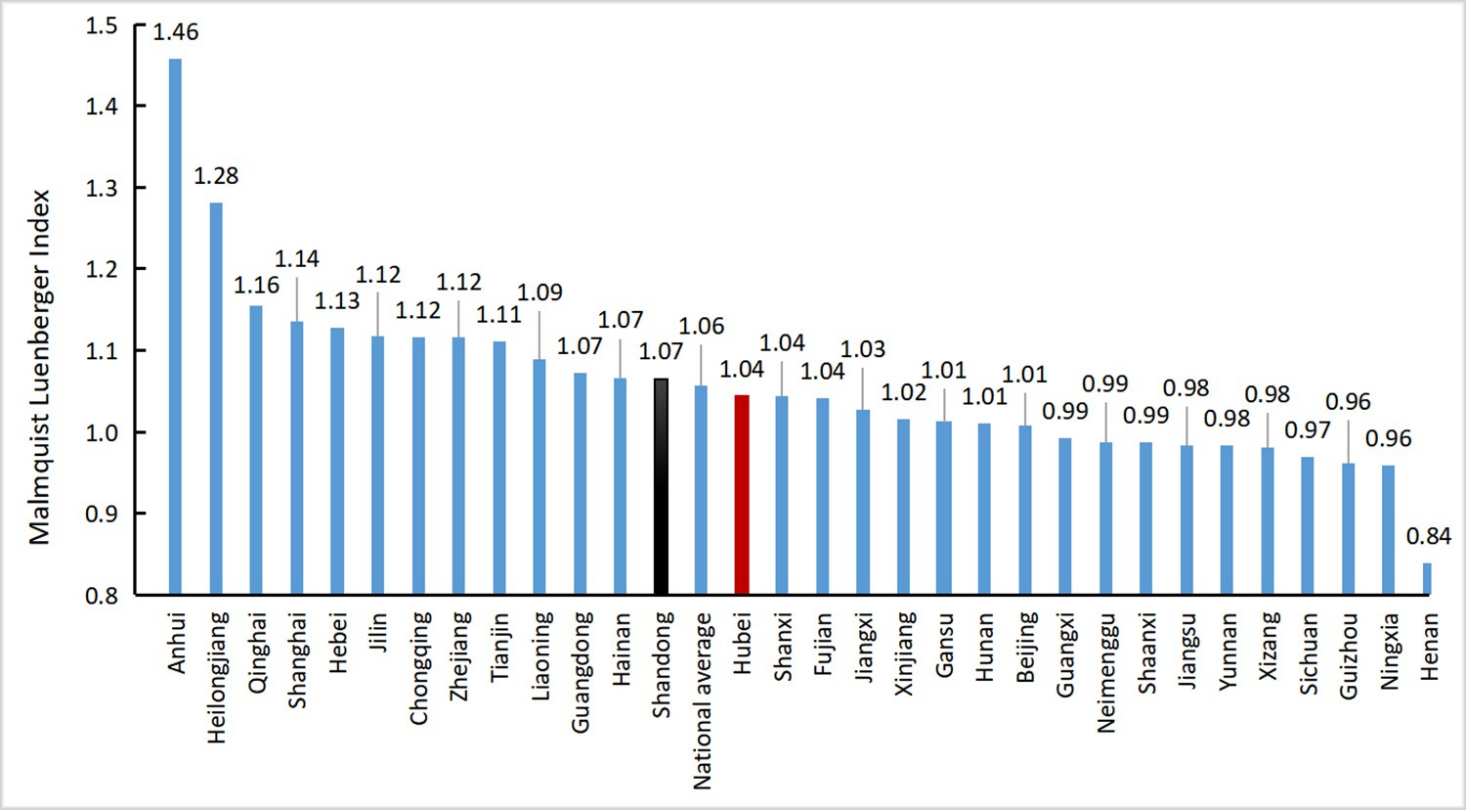
Histogram of ML index for 31 provinces.

The Malmquist–Luenberger index model in the DEA-SOLVER-PRO13 software was used to calculate and analyze the panel data of agricultural production in 31 provinces from 2004 to 2018. Table 3 presents the results. However, to reflect the agricultural carbon emission efficiency of each province more intuitively, a histogram of the average ML index of each province was drawn, as shown in Fig 4.

**Table 3:** Average values of ML index, *Effch* index and *Tech* index of 31 provinces.

Province	Average value			Province	Average value		
	*Effch*	*Tech*	ML		*Effch*	*Tech*	ML
Anhui	1.459	0.957	1.458	Gansu	1.017	1.010	1.012
Heilongjiang	1.085	1.169	1.281	Guizhou	1.017	0.955	0.961
Shanghai	1.175	1.014	1.136	Yunnan	1.035	0.962	0.983
Hebei	1.032	1.115	1.128	Guangdong	1.006	1.065	1.072
Jilin	1.034	1.162	1.117	Sichuan	0.996	0.971	0.969
Zhejiang	1.002	1.111	1.116	Shaanxi	1.015	1.095	0.987
Tianjin	1.071	1.069	1.110	Hunan	1.049	0.993	1.011
Liaoning	0.998	1.093	1.090	Chongqing	1.045	1.064	1.116
Shandong	0.998	1.068	1.065	Xinjiang	1.006	1.009	1.016
Shanxi	1.074	1.005	1.044	Guangxi	0.993	1.017	0.992
Fujian	1.008	1.031	1.041	Qinghai	1.126	1.003	1.155
Jiangxi	1.045	0.981	1.028	Ningxia	1.005	1.039	0.959
Beijing	1.016	0.996	1.008	Hubei	1.022	1.025	1.044
Neimenggu	0.994	0.992	0.988	Hainan	1.012	1.054	1.066
Jiangsu	0.995	0.989	0.983	Tibet	0.994	0.997	0.981
Henan	0.962	0.880	0.839	The whole country	1.041	1.029	1.057

It could be seen that in recent years, the national average agricultural carbon emission efficiency value was 1.057, and the ML index of 21 provinces in China was greater than 1, accounting for 68%. It was indicated that due to the success of low-carbon agriculture measures, most provinces had higher agricultural carbon emission efficiency. Thirteen provinces were above the national average level for agricultural carbon emissions. Among them, Anhui Province had the largest carbon emission efficiency at 1.458. This showed that the province had relatively perfect agricultural technology and a relatively high resource utilization rate, which could be used for reference by other provinces. Low-ranking Sichuan, Guizhou, Ningxia, Henan, and other places had relatively low agricultural carbon emission efficiency.

**Fig 5:**
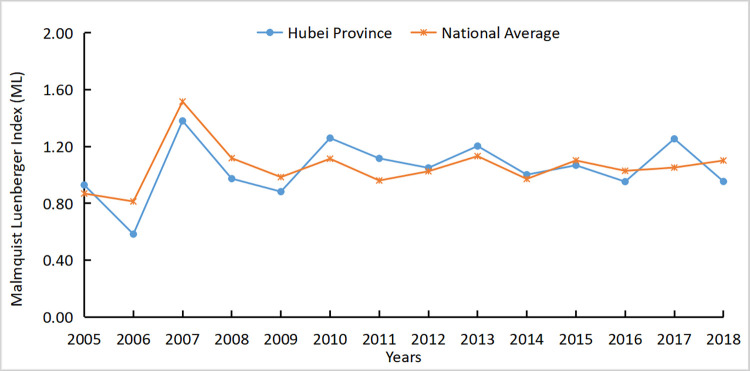
ML index of Hubei Province and national average from 2004 to 2018.

**Fig 6:**
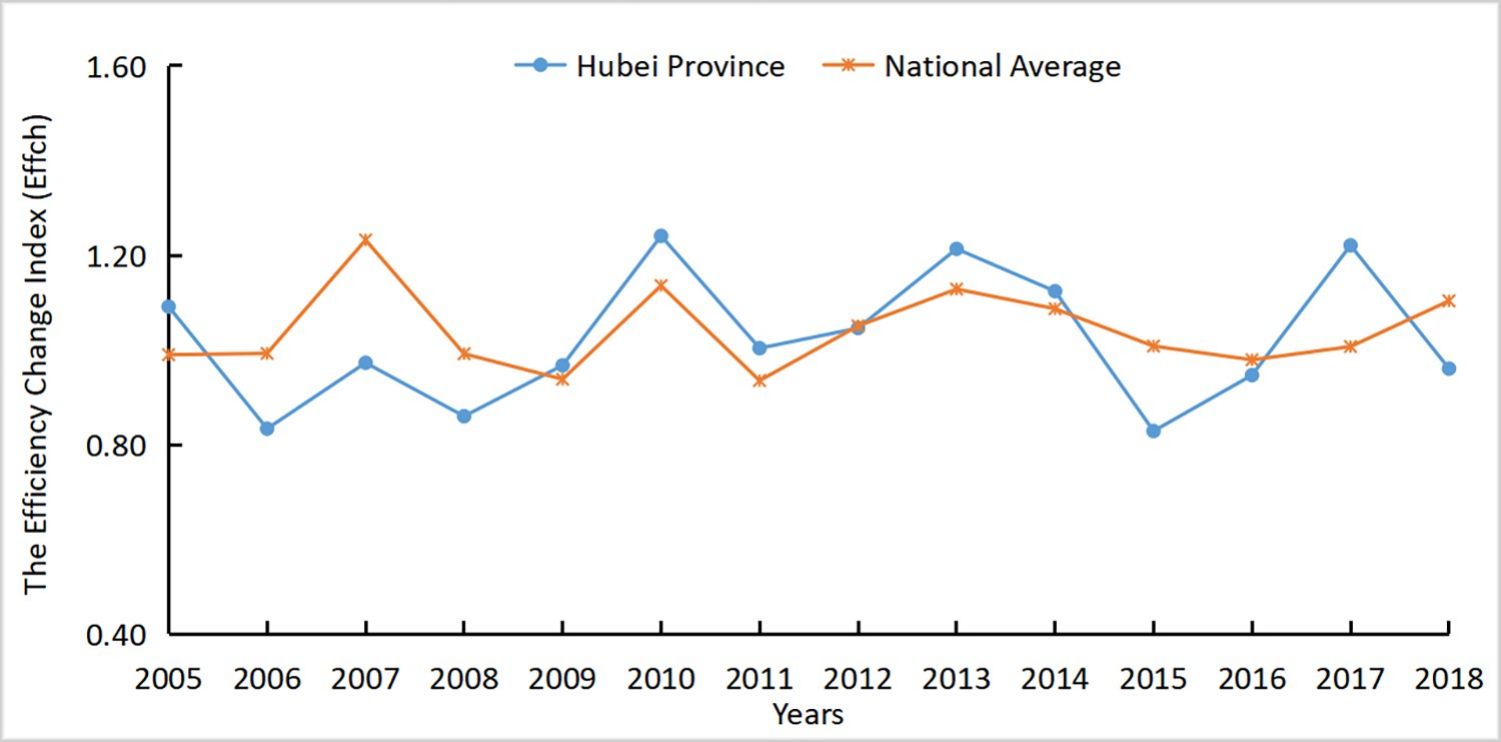
*Effch* index of Hubei Province and national average from 2004 to 2018.

**Fig 7:**
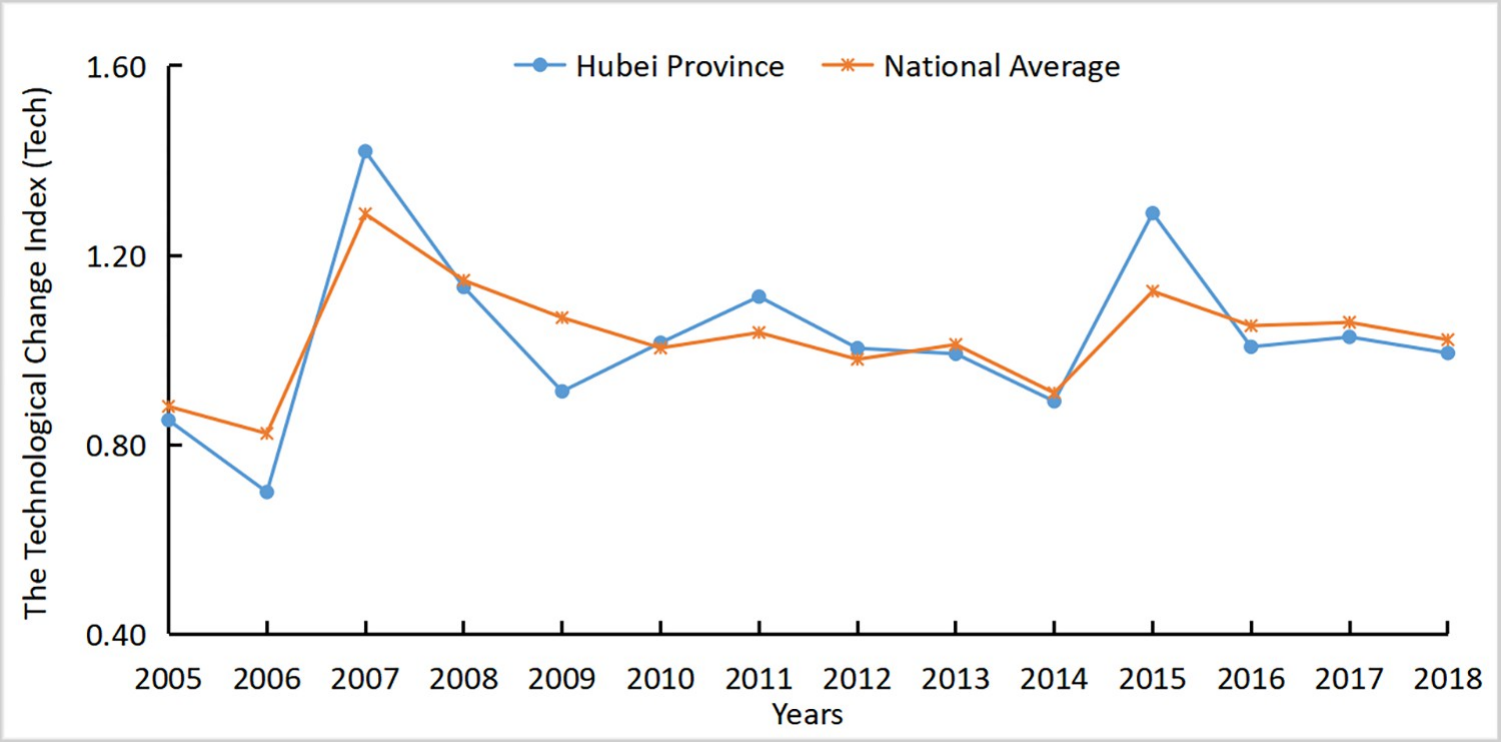
*Tech* index of Hubei Province and national average from 2004 to 2018.

The agricultural carbon emission efficiency of Hubei Province was 1.044, which is slightly lower than the national average of 1.057. The results showed that the carbon emission efficiency of Hubei Province was relatively high, but there is still room for further decrease. Figs 5–7 respectively reflected the changes in the agricultural carbon emission efficiency ML index, technical progress index, and efficiency change index in Hubei Province and the national average from 2004 to 2018.

It is apparent that from 2004 to 2009, the efficiency of agricultural carbon emissions in Hubei Province was slightly lower than the national average, while from 2009 to 2018, the efficiency of agricultural technology of the national average and Hubei Province alternately led. This shows that the agricultural technical efficiency in Hubei Province was generally rising but was slightly floating. According to the decomposition of the carbon emission efficiency index, the trend of the Hubei agricultural Tech index was consistent with the national agricultural Tech index from 2004 to 2018 but slightly lower than the national agricultural Tech index. After 2010, the Tech index and ML index of Hubei Province were greater than 1, and the two trends tended to be the same, showing agricultural technological progress provided a positive impetus for agricultural carbon emission efficiency. From 2005 to 2007, the Tech and ML indices increased by approximately 0.7. It indicated that agricultural technology was rapidly developed and popularized in this period, and the relative carbon emissions were controlled.

## 4 Conclusion

Agricultural carbon emissions were selected as an unexpected output, and an unexpected output SBM model was built to quantitatively analyze the efficiency of agricultural carbon emissions in Hubei Province from 2004 to 2018 in this study. Subsequently, the ML index was used to analyze the role of technological progress and technological efficiency changes in the development of low-carbon agriculture in Hubei Province.

The results show that during the study period, agricultural carbon emissions in Hubei Province increased first and then decreased. Agricultural production efficiency has improved, and the overall level has been slightly higher than the average level in China. Agriculture does not eliminate the extensive development model of high input, low efficiency, high emissions, and high pollution. The agricultural technological progress efficiency of agricultural resource utilization in Hubei Province was close to the optimal level and the improvement space was small. The low efficiency of agricultural technology was the key factor restricting the improvement of agricultural production efficiency in Hubei Province. The provinces in southern China, such as Fujian Province and Guangdong Province, had the highest efficiency, which is the benchmark for Hubei Province to improve agricultural production efficiency in the future. The key to improving the carbon emission efficiency of Hubei Province was the improvement of agricultural technical efficiency brought about by modernization.

## 5 Strategies and suggestions

The main factor for the increase in agricultural carbon emissions in Hubei Province was the scale of agriculture. Therefore, sustainable agriculture should be promoted with resource-saving, environment-friendly, and ecological conservation goals with the premise of ensuring food security. For example, increasing the popularization and use of water-saving irrigation facilities, encouraging the application of organic manure and green manure, reducing the use of chemical fertilizers, popularizing green prevention and control technology for crop diseases and insect pests, and replacing traditional agricultural production factors with low-carbon agricultural machinery to reduce agricultural carbon emissions.

Improving agricultural production efficiency, improving the farmland circulation system and promoting intensive use of farmlands, can vigorously develop agricultural scale, mechanize, and reforming agricultural low-carbon production technologies. By promoting and providing technical guidance to farmers, such as subsidies for new agricultural machinery and demonstration of soil testing and formula fertilization, modernization and low-carbon agricultural development can be realized and thus promote the transfer of the agricultural labor force to secondary and tertiary industries.

The development of modernization and mechanization of agriculture has resulted in a large surplus of the agricultural labor force. The transfer of the agricultural labor force can not only alleviate employment pressure but also further restrain agricultural carbon emissions. There is a need to develop the secondary and tertiary industries in townships and carry out training activities for agricultural labor force transfer and employment to absorb the surplus agricultural labor force in addition to optimizing the agricultural production structure.

According to the policy of structural reform on the supply side of agriculture, we should make full use of the advantages of various regions, adjust, and optimize the agricultural industrial structure, promote the quality and efficiency of agriculture, and promote low-carbon and efficient development of agriculture in various regions.

Additionally, improving the agricultural carbon emission regulatory system, making full use of the carbon emission inventory in Hubei Province, developing an independent agricultural carbon emission data information management and early warning system, and forecasting and monitoring agricultural carbon emissions dynamically in real-time are some key areas. Counties and cities should prepare annual reports on agricultural carbon emission data and establish a long-term supervision mechanism as well as establish a sound agricultural ecological compensation mechanism to promote the orderly development of low-carbon and high-quality agriculture in Hubei Province.
